# Fungal Keratitis in a Tertiary Hospital in Malaysia

**DOI:** 10.7759/cureus.18389

**Published:** 2021-09-29

**Authors:** Chow Tze-Suen, Tan Chew-Ean, Norshamsiah Md Din

**Affiliations:** 1 Department of Ophthalmology, Hospital Sultanah Bahiyah, Alor Setar, MYS; 2 Department of Ophthalmology, Universiti Kebangsaan Malaysia Medical Centre, Kuala Lumpur, MYS

**Keywords:** fungus, evisceration, penetrating keratoplasty, fungal keratitis, corneal ulcer

## Abstract

Background

Fungal keratitis is one of the commonest causes of corneal blindness in developing countries including Malaysia. We aim to evaluate the sociodemographic background, clinical features, predisposing factors, laboratory findings, management, complications, and visual outcome of patients with fungal keratitis in a tertiary hospital in Malaysia.

Methods

A retrospective review of medical and microbiology records of all patients treated for corneal ulcer from 2015 to 2019 at Hospital Sultanah Bahiyah was performed. Sociodemography, predisposing risk factors, clinical characteristics, causative organisms, and final visual outcome were analyzed.

Results

We identified 103 patients with a diagnosis of fungal keratitis. The majority of the patients were of Malay ethnicity (85.4%) and male gender (81.6%), with an age range of 21 to 60 years (94.1%). Ocular trauma was the main predisposing factor in 82 (79.6%) patients. Poor visual improvement was observed in groups with ulcer more than 4mm (67.5%), presence of hypopyon (50.9%), and high intraocular pressure (75.0%) upon presentation. *Fusarium *spp. (19.4%) was the commonest fungus isolated followed by *Aspergillus *spp. (5.8%). All patients were prescribed either topical, oral, intracameral, or combined therapy, whereas 20 (19.4%) patients required surgical intervention, of which 16 (15.5%) underwent penetrating keratoplasty and three (2.9%) required evisceration.

Conclusion

The epidemiological, socioeconomic, and predisposing factors may facilitate timely diagnosis and prompt treatment to achieve a better visual outcome and minimize complications including corneal blindness.

## Introduction

Fungal keratitis is one of the commonest causes of corneal blindness, contributing to 24.8% of all corneal ulcer cases in Malaysia [1]. The World Health Organization (WHO) found out that 4% cases of all blindness are caused by corneal opacities [1]. Several predisposing factors were identified, including ocular trauma, prolonged use of topical steroid, underlying ocular surface disease, contact lens wear, diabetes mellitus, and immunosuppression. Malaysia with its warm climate and high humidity year-round provides a conducive environment for sporadic fungal growth. Hence, high clinical suspicion is vital even if there is no significant history of trauma by a vegetative matter or direct inoculation [2,3].

Clinical manifestations supported by corneal scraping microscopy examination and culture remain the mainstay of diagnosis. *Fusarium* spp., *Candida* spp., and *Aspergillus* spp. are frequently found in the cultures from corneal scraping [4]. Because of the potential delay in culture confirmation, early initiation of antifungal treatment should be based on clinical judgment to achieve a better outcome and minimize devastating complications such as corneal scarring, corneal perforation, and endophthalmitis [2,4].

We aim to report the sociodemographic background, clinical features, predisposing factors, laboratory findings, management, complications, and visual outcome of patients in a tertiary hospital in Malaysia. This information will allow the profiling of fungal ulcers and facilitate early diagnosis and prompt treatment in future cases.

## Materials and methods

This is a retrospective review of patients diagnosed with fungal keratitis in Hospital Sultanah Bahiyah, a tertiary referral center in northern Malaysia. This study was granted ethical approval by the Malaysian National Institute of Health and was registered in the National Medical Research Registry (NMRR-20-1473-55317).

All corneal scrape samples undertaken from January 2015 to December 2019 were extracted from electronic records. Patients with the diagnosis of fungal keratitis were identified and included in this study. The database was then used to retrieve patients’ demographic information, clinical comorbidities, predisposing factors, clinical features of fungal keratitis, laboratory findings, duration of hospitalization, treatments, complications, and visual outcome of the patients.

The comprehensive assessment included medical history, presenting visual acuity, slit-lamp biomicroscopic examination, and corneal scraping of the affected eye. Microscopic evaluation, which included gram stain and 10% potassium hydroxide (KOH) mount, was performed on collected corneal smears as part of the standard protocol. Other culture samples were sent using blood agar, chocolate agar, Sabouraud dextrose agar, and MacConkey agar.

Treatment given to patients was individualized based on clinical evaluation and laboratory findings. Routine follow-ups at regular intervals were conducted to assess the treatment efficacy, disease progression, and possible complications. Final visual acuity was taken at least six months after completion of treatment. Visual acuity was recorded using the Snellen chart, grouped as good (6/6 to 6/12), moderate (6/15 to 6/60), and severe (worse than 3/60) [5]. Patients with incomplete data were excluded from this study.

Statistical analysis for descriptive analysis was performed using SPSS 22.0 (IBM Corp., Armonk, NY). Pearson’s chi-square test was used to determine the relationship between two categorical data. A p-value of less than 0.05 was considered statistically significant.

## Results

A total of 103 patients with the diagnosis of fungal keratitis were enrolled in this study. The mean age was 51.1±17.1 years (range: 19-87 years). The majority of patients were male (n=84, 81.6%), while 19 (18.4%) were female. Majority of them Malay ethnicity (85.4%), followed by Chinese (6.8%), non-Malaysian (6.8%), and Indian (1.0%). Only 15 (14.6%) smokers were recorded, while 17 (16.5%) patients had diabetes mellitus (Table 1).

**Table 1:** Sociodemographics of patients

Demographic variables	Frequency (n)	Percentage (%)
Age (years)		
<21	1	1.0
21–40	37	35.9
41–60	29	28.2
61–80	31	30.1
>80	5	4.9
Gender		
Male	84	81.6
Female	19	18.4
Ethnicity		
Malay	88	85.4
Chinese	7	6.8
Indian	1	1.0
Others/foreigners	7	6.8
Smoker		
Yes	15	14.6
No	88	85.4
Diabetes mellitus		
Yes	17	16.5
No	86	83.5

Our findings showed that ocular trauma was the major predisposing ocular factor (82, 79.6%), followed by corneal suture related in six (5.8%) patients, contact lens related in four (3.9%) patients, bullous keratopathy in three (2.9%) patients, ocular surface disease in two (1.9%) patients, and no known predisposing factor in six (5.8%) patients. Among those with ocular trauma, 29.1% were due to vegetative matter as compared to 27 (26.2%) cases caused by non-vegetative matter such as stone, sand, dust, metal, chemical, and insect (Table 2).

**Table 2:** Predisposing ocular factors for fungal keratitis

Predisposing ocular factors	Frequency (n)	Percentage (%)
Ocular trauma	82	79.6
Vegetative	30	29.1
Non-vegetative	27	26.2
Unknown	25	24.3
Ocular surface disease	2	1.9
Contact lens related	4	3.9
Bullous keratopathy	3	2.9
Corneal suture related	6	5.8
None	6	5.8

Table 3 showed the final visual acuity in our patients in relation to various clinical features. Large ulcer diameter (p<0.001), central location of the fungal ulcer (p=0.006), presence of hypopyon (p=0.002), and high intraocular pressure (IOP) (p<0.001) were significantly associated with worse final visual acuity. The duration of symptoms prior to presentation had no important association with visual outcome (p>0.05) (Table 3).

**Table 3:** Presenting and final visual acuity based on clinical features *Visual acuity: good, 6/6–6/12; moderate, 6/15–3/60; severe, <3/60 ^Ɨ^Pearson’s chi-square test: p < 0.05 is significant

	Final visual acuity*, n (%)			p-Value^Ɨ^
	Good	Moderate	Severe	
Presenting interval (days)				
≤3	23 (42.6)	11 (20.4)	20 (37.0)	0.114
>3	13 (26.5)	18 (36.7)	18 (36.7)	
Size, largest diameter (mm)				
≤2	17 (65.4)	4 (15.4)	5 (19.2)	<0.001
2–4	16 (43.2)	15 (40.5)	6 (16.2)	
>4	3 (7.5)	10 (25.0)	27 (67.5)	
Location				
Central	5 (15.6)	7 (21.9)	20 (62.5)	0.006
Paracentral	24 (43.6)	18 (32.7)	13 (23.6)	
Peripheral	7 (43.8)	4 (25.0)	5 (31.3)	
Hypopyon				
Present	13 (22.8)	15 (26.3)	29 (50.9)	0.002
Absent	23 (50.0)	14 (30.4)	9 (19.6)	
Intraocular pressure (mm Hg)				
10–21	36 (43.3)	24 (28.9)	23 (27.7)	<0.001
>21	0 (0.0)	5 (25.0)	15 (75.0)	

Out of 103 cases, causative fungi were successfully isolated in 41 (39.8%) cases, in which *Fusarium* spp. was mostly yielded (20, 19.4%) followed by *Aspergillus* spp. (n=6, 5.8%) (Table 4).

**Table 4:** Causative fungus, antifungal prescribed, and surgical Intervention

	Frequency (n)	Percentage (%)
Causative fungus		
*Fusarium* spp.	20	19.4
*Aspergillus* spp.	6	5.8
*Curvularia* spp.	3	2.9
Non-sporulating hyaline hyphomycetes	3	2.9
*Candida* spp.	2	1.9
*Bipolaris* spp.	2	1.9
*Nigrospora* spp.	2	1.9
*Chrysosporium* spp.	1	1.0
Unidentified	62	60.2
Antifungal prescribed		
Single topical antifungal	9	8.7
Dual topical antifungals	40	38.8
Combined topical and oral antifungals	44	42.7
Topical, oral, and intracameral antifungals	10	9.7
Surgical intervention		
Penetrating keratoplasty	16	15.5
Evisceration	3	2.9
Corneal glue with bandage contact lens	3	2.9
Tarsorrhaphy	1	1.0

The treatment regime was decided based on clinical manifestation, progression, and response to treatment. Most (44, 42.7%) patients were treated with combined topical and oral antifungals, followed by dual topical antifungals (40, 38.8%), combined topical, oral, and intracameral antifungals (10, 9.7%), and single topical antifungal (9, 8.7%) (Table 4).

A total of 20 (19%) patients had to undergo surgical intervention including penetrating keratoplasty (PK) (n=16, 15.5%), evisceration (n=3, 2.9%), corneal glue with bandage contact lens (n=3, 2.9%), and tarsorrhaphy (n=1, 1.0%) (Table 4).

## Discussion

Fungal keratitis is a great public health threat especially in developing countries as it is one of the major causes of corneal blindness in a relatively younger age group [4,6]. The annual global incidence of fungal keratitis is 23.6 per 100,000 population but this figure is particularly higher in Asia mainly in the southern and south-eastern regions, with an incidence of 73 and 15 cases per 100,000 population, respectively [4,6]. This can be attributed to the warm weather and high humidity, as well as agricultural and outdoor work as main economic activities in these regions [6]. Our study recorded an annual incidence of five cases per 100,000 population, which is higher than Malaysia’s national average incidence of 1.3 per 100,000 population [6].

Kedah is a state in northern Malaysia where agriculture is the main economic activity. All these geographic and socioeconomic properties are the main contributing factors to a higher incidence of fungal keratitis in our population. Local studies in other states of Malaysia with a similar background supported the findings in our study [3,4,5,7]. Besides that, we also discover the relevance of the abovementioned factors in the higher incidence of fungal keratitis in India and China [1,5,8,9].

The patient profile in our study found that most of them were Malay (85%), male (82%), and young (65% aged 21 to 60 years). Ocular trauma resulted in almost 80% of cases, predominantly caused by vegetative matters (30, 29.1%). This conforms with the local sociodemographic profile in which Malay is the major ethnicity and young males are the main workforce in a largely agricultural state, including farmers, fishermen, and laborers. Our results are in line with several other local studies [1,2,5]. Koh et al. found that diabetes mellitus was a common risk factor for corneal ulcer due to impaired corneal barrier function and delayed epithelial healing [10]. However, no significant relationship can be established between smokers or diabetic patients with fungal keratitis in our study.

Visual acuity was assessed using the Snellen chart at the first presentation and subsequent visits. Final visual acuity was assessed at least six months from the completion of optimal treatment. Delayed presentation and treatment are known to be associated with poorer visual outcome. Around 47.5% of patients in our study presented more than three days from the onset. Factors contributing to the delayed presentation included lack of awareness, self-remedy, and over-the-counter drugs used. Our study demonstrated that large ulcer diameter (> 4mm), central fungal ulceration, presence of hypopyon, and high IOP (> 21 mmHg) on presentation were poor prognostic factors for the final visual outcome. Our study findings were parallel with studies conducted by Chitamparam et al. and Prajna et al. [7,11]. Central fungal ulceration disrupted the visual axis, resulting in worse final vision as compared to paracentral and peripheral ulceration [7].

*Fusarium* spp., *Aspergillus* spp., and *Candida* spp. are known to be the commonest causative fungi in mycotic keratitis [6]. Our study revealed *Fusarium* spp. was the main causative fungus (19.4%) followed by *Aspergillus* spp. (5.8%). Yap et al., Mohd-Tahir et al., and Chitamparam et al. also demonstrated *Fusarium* spp. as the commonest causative organism in fungal keratitis [1,2,7].

Most of our patients received either dual topical antifungal (amphotericin B and fluconazole) or combined topical and oral antifungal (fluconazole). Only 10% of our patients required combined topical, oral, and intracameral antifungal. Cochrane review suggested that natamycin was superior in treating *Fusarium* fungal ulcers compared to other antifungal agents. However, natamycin is expensive and is not routinely available in Malaysia due to its cost [12]. Sharma et al. showed that there was no significant extra benefit of intracameral amphotericin B over topical therapy in the Indian population [13].

Surgical treatment is indicated in cases refractory to medical therapy and deep or severe fungal keratitis [14]. Twenty (19.4%) patients from our study had to undergo surgical intervention such as PK and evisceration. The surgical rate in our study is almost similar to another local study in Kelantan by Mohd-Tahir et al. who showed that 19.5% of patients required PK and 4.3% required evisceration [2]. A study carried out in North China recorded 37.8% of PK was performed in their patients due to delay in presentation and wrong usage of drugs received prior to admission [14]. PK remains the commonest surgical intervention with promising improvement in visual outcome [15,16].

There are several limitations to our study. Due to the retrospective nature and incomplete medical records, sociodemographic information such as occupation, education level, and household income could not be evaluated. Efficacy of different treatment regime is also incomparable due to the retrospective nature of this study and the lack of standardized treatment protocol. In any case, the existing data may help identify commoner causative organisms based on the clinical features and predict the patients’ final visual outcome.

## Conclusions

Ocular trauma was the main predisposing factor in our study due to high involvement in agricultural and outdoor activities. Bigger ulcer size, central corneal involvement, presence of hypopyon, high IOP, and poor vision at presentation are factors for poorer final visual outcome. A high index of suspicion of fungal keratitis especially when treating patients with known predisposing factors facilitates early diagnosis and prompt treatment, preventing unfavorable visual outcome.
